# Resilience Competence Face Framework for the Unforeseen: Relations, Emotions and Cognition. A Qualitative Study

**DOI:** 10.3389/fpsyg.2021.669904

**Published:** 2021-06-23

**Authors:** Marius Herberg, Glenn-Egil Torgersen

**Affiliations:** ^1^Department of Military Leadership and Sport Science, Norwegian Defense University College, Oslo, Norway; ^2^Department of Psychology, Norwegian University of Science and Technology, Trondheim, Norway; ^3^USN School of Business, Department of Business, History and Social Sciences, Center for Security, Crisis Management and Emergency Preparedness, University of South-Eastern Norway, Notodden, Norway

**Keywords:** resilience, competence, emotions, relations, sensemaking, the unforeseen, crisis management, organizational learning

## Abstract

The high impact of unforeseen events in a globalized world accentuates the importance of a greater in-depth and broader understanding of resilient competencies that can promote performance. Traditional research has, however, paid relatively little attention to uncertainty and unpredictable conditions, including the particulate competence of the unforeseen, and how organizations can achieve degrees of resilience. Hence, the purpose of this study is to explore whether there are types of competence at the individual, social and organizational level that can enhance preparedness to face the unforeseen. The first aim was to explore how highly experienced professionals from different sectors and organizational levels describe and understand the nature and function of the unforeseen phenomenon. The second aim was to explore what resilient competencies can be beneficially applied in organizations to enhance performance irrespective of the scenario or event that occurs. The generic qualitative approach of this study employed semi-structured interviews. The purposive expert sample of 13 highly knowledgably Norwegian professionals with unique and extensive cross-sectorial experience of unforeseen events were selected. Ages ranged from 41 to 62 years (*M* = 48.92, *SD* = 6.94), length of professional experience and education ranging from 22 to 43 years. Thematic analysis of interview transcripts and the interpretation displayed six types of resilience competence: *(1) General Preparedness, (2) Characteristics and Competence of the Individual, (3) Sound Relations, (4) Creative Behavior and Improvisational Skills, (5) The Ability to Reflect and Learn, (6) Emotion Efficacy*. In addition, *The Unforeseen* was discerned as a complex phenomenon. These findings emphasize a cross-disciplinary perspective and provides integrative multilevel insight into the particulate competence of the unforeseen by introducing a framework that serves as a foundation for future research and as a tool for practitioners working in the field.

## Introduction

*Between stimulus and response there is a space. In that space is our power to choose our response. In our response lies our growth and our freedom*. Viktor E. Frankl

Many people and organizations across the globalized world experience unforeseen events, including crisis and emergency responders. How do these professional experts experience, adapt to and manage new threats in a volatile, uncertain, complex and ambiguous environment (VUCA) (Barber, 1992; Bennett and Lemoine, 2014; Antonacopoulou et al., 2019)? The COVID-19 pandemic, foreseeable, yet largely unforeseen, has shown us that a small virus can impact the global community so fast and with major consequences. The high impact of events such as this accentuates the importance of a more in-depth and broader understanding of performance, capabilities and entangled types of resilience competence that can promote preparedness for the unforeseen (Bhamra et al., 2011; Boin and van Eeten, 2013; Seville, 2016; Torgersen, 2018a; Hollnagel, 2021). This study aims to provide insight into these issues, based on semi-structured interviews with 13 highly knowledgably professionals from different sectors and organizations, primarily in Norway. Traditional research within crisis management and resilience has mainly been system-orientated and used separate and aggregate concepts of competence (Bundy et al., 2017; Williams et al., 2017; Stokes et al., 2019). The overall purpose of this study is therefore to supplement this research with a cross-disciplinary perspective, and to present integrative multilevel knowledge into the particulate competence of the unforeseen.

Most organizations, however, appear to learn from repeated successes rather than from exceptional events or failure (Starbuck, 2009, p. 934). Preparing and learning from highly infrequent and unknown events therefore appears to represent a contradiction (Lanir, 1983; Lampel et al., 2009). These events can range from life crises that affect individuals, to major global and nationwide extreme events such as pandemics, natural disasters, cyber-attacks, and terrorist attacks (Westrum, 2006). The risk level is also often high, the events manifesting suddenly as discontinuities, disorder and disruptions of routine (Ansell et al., 2010; Lu and Xue, 2016). Dealing with the unknown, unpredictable and uncertainty obviously presents educational and learning challenges (Fischhoff, 1975; Barnett, 2004; Garvin et al., 2008). Many organizations consequently struggle to anticipate and prepare for unforeseen events, to monitor, learn and respond quickly to change and to maintain an appropriate degree of fit with the environment (Lagadec, 1993; Cunha et al., 1999; Hollnagel, 2015, 2021). Different countries and sectors also have different policies, structures and organizational approaches to emergency and crisis management (Kuipers et al., 2015).

Previous studies indicate that it is possible to prepare and ensure adaptive responses to unforeseen and extreme events, by implementing measures that improve social resources such as interaction, cognition, leadership, trusting relationships, cohesion, well-being, social support, learning-oriented culture, and creative behavior (Bliese and Britt, 2001; Bass et al., 2003; Comfort, 2007; Hannah et al., 2009; Campbell et al., 2010; Kuntz et al., 2016; Torgersen, 2018b; Williams et al., 2019). Studies also show that an individual’s psychological resources such as personality, mental abilities, traits, resilience, and self-efficacy appear to be beneficial when dealing with stress, challenges and adversity (Lazarus and Folkman, 1984; Luthans et al., 2007; Maddi, 2007; Hannah et al., 2008; Eschelman et al., 2010; Delahaij et al., 2016; Larsen et al., 2017). Furthermore, important organizational and operational resources in the preparedness for the unforeseen include contingency plans, emergency exercise, available materials, mastery of equipment, improvisation and flexibility (Bechky and Okhuysen, 2011; Hadida et al., 2015; Haddow et al., 2017; Herberg et al., 2019). This therefore suggests the importance of a multivariate, multilevel and interdisciplinary approach to the identification of competencies that can affect the ability to face unforeseen events.

The unforeseen is used in this study as a collective term for a number of seemingly similar expressions such as the “unexpected,” “uncertain,” “unpredictable,” “unthinkable,” and the “unlikely” (Torgersen, 2015). An unforeseen event is *a relatively unknown event or situation that occurs relatively unexpectedly and with relatively low probability or predictability to the individual, group or community that experience and manage the event* (Kaarstad and Torgersen, 2017, p. 1). The high degree of unpredictability and complexity associated with unforeseen events increases the need for an institutional apparatus that prepares for the full spectrum of stressors, threats and transboundary hazards (Comfort, 2007; Breakwell, 2014; Boin, 2019). It may be particularly important that organizations such as the health service, the police, the military, and the fire and rescue service comprehend and manage such events. Emergency preparedness and risk management typically focus on expected scenarios of known magnitudes (Pearson and Mitroff, 1993; Torgersen, 2018a). The real outcomes of this preparation are often, however, uncertain and unpredictable (Cunha et al., 2005).

It is important, despite the unexpected or unknown nature of disruptive events, that acceptable functioning is maintained and that ever-changing situations can be quickly adapted to and even benefited from (Hémond and Robert, 2012). The idea of resilience is therefore gaining ground in the field of crisis management (Boin and van Eeten, 2013; Williams et al., 2017). The concept of resilience can be traced to a variety of fields, taking its cue from systems theory (Matyas and Pelling, 2015), and worked out through socio-ecology (Holling, 1973), engineering (Hollnagel et al., 2008) and psychology (Meredith et al., 2011). The term is both multidisciplinary and multifaceted (Sage et al., 2014). Resilience is, furthermore, related to both the individual (Luthans et al., 2006; Britt et al., 2016) and organizational responses (Hamel and Välikangas, 2003; Sheffi, 2017) and involves both the ability to endure discontinuities and to adapt and exploit new contexts (Bhamra et al., 2011).

For the purpose of this study, the following definition of resilience is used; *the intrinsic ability of a system to adjust its functioning prior to, during, or following changes, disturbances, and opportunities so that it can sustain required operations under both expected and unexpected conditions* (Hollnagel, 2015, p. 25). This definition incorporates the dynamic aspects of change and learning across contexts, including a positive orientation to outcomes, and the strength to master and respond to adversity and stress. It has been suggested that resilience is a valuable antecedent of adaptability and transformability to discontinuities and the unexpected (Walker et al., 2004; Weick and Sutcliffe, 2015). The primary aim of this study is therefore to explore types of resilience competence at the individual, social and organizational level that can enhance performance irrespective of the scenario or event that occurs (Tasic et al., 2019).

Many academics, policymakers and professional practitioners consider human resources to be a unique internal and sustainable resource of strategic importance to the organization (Armstrong, 2011; Kramar, 2014; Armstrong and Taylor, 2020). It has therefore been argued that the competencies of the employees is a resilient capability, and is one that could gain a competitive advantage and enhance the performance of an organization (Guest, 1997; Wright et al., 2001, 2005).

Competence is defined in this study as being; *a wide resilient and relational concept which embodies the capability to transfer the combined knowledge, skills, abilities, and attitudes to meet demands or carry out tasks successfully under a variety of conditions, individually or with others. Competence encompasses sustainability in organization and planning of work, innovation and the capacity to comprehend and manage non-routine activities based on the internal and external standards, values, and objectives of the occupational area. It includes the relational qualities and the personal effectiveness required to interact with co-workers, managers, partners and citizens* (based on; Boyatzis, 1982; NVQ, 1994; OECD, 2002, p. 8; HSE, 2003; Lai, 2013). The concept of competence for the unforeseen is perceived as a panarchy set as three adaptive cross-level cycles, from an arching level to a more particular level (Holling and Gunderson, 2002; Torgersen and Steiro, 2009); the main type of competence, units of competence, and elements of competence (Burke, 1990; Truelove, 1995).

The strategic implication of this competence-based view is often expressed through the implementation of targeted activities and measures to increase the performance of the individual and the organization (Boyatzis, 2008; Armstrong and Taylor, 2020). Research into areas of competence that can facilitate performance in encounters with unforeseen events therefore has genuine practical value. Guidance on how organizations should target and develop resilient resources could also be developed, if the concept of competence for the unforeseen could provide knowledge on how individuals, groups and organizations perform.

It is essential for any organization to contain its existence, and be able to manage the conditions necessary to cope with the challenges and opportunities it faces (Hollnagel, 2021, p. 9). The limited body of literature on competence for the unforeseen indicates that it is possible to manage these events by implementing measures that are viewed as resilient core competencies (Herberg et al., 2018, 2019; Torgersen et al., 2018). Previous studies have, however, shown deficiencies and limitations in the research. For example, dominant and promising factors that could predict the outcome of such events are often not studied concurrently (Bonanno et al., 2010; Britt et al., 2016). The sample of organizations that have been studied is small. Research has primarily focused on a theoretical model of competence for the unforeseen which is based on certain variables (Herberg et al., 2018, 2019). There is also little research that empirically proves theories of how organizations achieve degrees of resilience (Bhamra et al., 2011). Many studies furthermore focus on predictable conditions, the outcomes thus not necessarily involving uncertainty and risk (Torgersen, 2015, 2018a; Geier, 2016). Not all the important variables are therefore known (Morse, 1991). There may be nuances, gaps or oversights that need to be filled (Pratt, 2009, p. 859). How organizations can cultivate the capabilities that can be beneficially applied in practice, should therefore be explored more methodically and scientifically.

The explicit purpose of this study is to explore how highly experienced professionals from different backgrounds, and with a unique experience of the unforeseen, comprehend, prepare, manage, and learn from these events. As such, this study seeks to empirically pursue previous findings, and to more closely examine and better understand the influence that individual, social and organizational factors have upon the meaning individuals ascribe to wide-ranging experiences of unforeseen events. New avenues also need to be explored and the complexity of the unforeseen phenomenon needs to be reported. This study therefore employs a generic qualitative research approach to address these multi-dimensional phenomena, and to allow simultaneous further exploration of relevant variables and their interrelations (Percy et al., 2015; Bradbury-Jones et al., 2017).

Two main research questions were posed:

(1) How do highly experienced professionals from different sectors describe and understand the nature and function of the unforeseen phenomenon?
(2) What competencies at the individual, social and organizational level can enhance readiness and preparedness to face unforeseen events?

## Materials and Methods

### Procedure

This study explores data collected from qualitative interviews with experienced professional experts (Creswell and Creswell, 2018). The qualitative research approach was used to gain new, more in-depth, detailed, and rich information that can enhance our understanding of the unforeseen phenomenon and the competence and complex relations of handling such events (Morse and Richards, 2002; Ritchie and Lewis, 2003). Previous research has given us some pre-knowledge within these issues. We, however, wanted to more fully describe and explore experiences in unforeseen events from the perspective of highly knowledgeable persons (Aronson, 1994; Percy et al., 2015). The qualitative research approach allowed us to further explore previously developed theory and to bridge the gap between theory and practice (Thorne, 2008), based on the interrelated concepts of interpretivism and on reflexivity balanced with a pragmatic worldview (Smith et al., 2011, p. 9; Creswell and Creswell, 2018). A generic qualitative design and interview data collected from a highly informed purposeful sample were therefore consistent with the purpose of the study (Merriam, 2002; Caelli et al., 2003; Kahlke, 2014; Percy et al., 2015; Bradbury-Jones et al., 2017). Moreover, thematic analysis was considered as the method to best suit the research questions posed, and for elucidating the participants’ experiences, reflections, views and conceptualizations of the phenomenon and competence for the unforeseen (Boyatzis, 1998; Braun and Clarke, 2006, 2013; Joffe, 2012). The individual interviews were conducted between January and April 2019 in Norway.

### Participants

The study participants were 13 influential and well-informed Norwegian leaders and experts with unique and extensive cross-sectorial professional experience of unforeseen events. Table 1 shows the characteristics of the participants. Table 2 shows examples of unforeseen events. This study used purposive sampling procedures to provide a wide range of perspectives and information-rich cases on the topic (Patton, 2002; Percy et al., 2015). 17 persons were approached via email. One was unavailable, two did not respond, and one was left out as saturation was sufficient. Eleven were male and two were female. Ages ranged from 41 to 62 years (*M* = 48.92, *SD* = 6.64), length of professional experience and education ranging from 22 to 43 years. The number of participants was not set in advance. Sampling for this study was instead conducted until a satisfactory diversity of perspectives, roles and functions was achieved, and until the contribution of each interview became negligible (thematic saturation) (Morse and Richards, 2002; Patton, 2002).

**TABLE 1:** Matrix of study participants.

**P**	**Role or function and position**	**Sector or industry**	**Experience and organizational level**			**Age**	**Gender**
			**Strategic/political**	**Operational/staff**	**Operative/tactical**		
1	Security Advisor Middle/Operational level	Private security industry Previous Police	(x)	**x**	x	61	M
2	Unit Leader Middle/Operational level	Civil Defense	x	**x**		54	F
3	Operator/Unit Leader Middle/Operative level	Police		x	**x**	44	M
4	Security Advisor Middle/Operative level	Energy industry Multinational Prev. Military	(x)	**x**	x	45	M
5	Unit Commander Top level	Energy industry Multinational Prev. Police	**x**	x	(x)	45	M
6	Senior Advisor Top level	The telecom industry Multinational Prev. Public sector	**x**	x		43	M
7	Unit Commander Top level	Military	**x**	(x)	x	50	M
8	Staff/Operator Middle/Operative level	Health service Military		**x**	x	49	M
9	Senior Advisor Top level	Military	x	**x**	(x)	51	F
10	Unit Commander Middle level	Fire service	(x)	**x**	x	41	M
11	Group President Top level	Consumer Products Multinational	**x**	x		47	M
12	Senior Consultant Top level	Private counseling and security industry Prev. Military	**x**	x	(x)	62	M
13	Operator/Unit leader Operative level	Fire service		x	**x**	44	M

**P, participant; M, male; F, female.****x***, *primary experience; x*, *secondary experience; (x)*, *tertiary experience. Selection criteria; (a)*, *unique experience in dealing with unforeseen events; (b)*, *experience in different roles, highly knowledgeable; (c)*, *high level of competence (expert, master level); (d)*, *represents different organizational and competence levels, functions, and role; (e)*, *cross-sectoral (Military, Police, Health service, Fire service, Private sector); (f)*, *good communication skills, open and honest. (g)*, *demographic criteria (age, gender).**

**TABLE 2:** Examples of unforeseen events experienced by the study participants.

Specific events*^1^	**Events of general character**
Avalanche accident Vassdalen, Norway, 1986	Mentally unstable persons, accidents, illness, suicide and sudden death
Tsunami, Thailand, 2004	Crime and violent incidents/situations
Financial crisis, worldwide, 2008	Fire incidents
Fire in the Oslofjord subsea tunnel, Norway, 2011	Cyber-attacks
Terrorist attacks on Oslo and island of Utøya – 22 July, Norway, 2011	Major restructuring and changes of organizations in crisis
Terror attack and hostage situation In Amenas, Algeria, 2013	Natural disasters such as earthquakes (e.g., Afghanistan and Nepal), forest fires, floods, power outflows and ash cloud
The frigate HNoMS*^2^ Helge Ingstad Collision with oil tanker, Norway, 2018	Military operations in war and conflict areas such as Afghanistan, the Balkans, Lebanon from the 1990s to the 2010s
	Personal or professional betrayal and infidelity
	Media leaks and media matters

*^1^Description, place, year. *^2^HNoMS, His/Her Norwegian Majesty’s Ship. *Participants’ experiences with unforeseen events ranged from minor personal everyday crises to medium and large national and international events. The events the participants reported varied from the personal, the group, the organizational, the cross-sectoral and the societal level.*

The participants were identified through personal networks and suggestions. Most were also well-known from the media or from seminars related to the topic of this study. They were recruited by a combination of email (to which an information sheet and declaration of consent were attached), SMS and social media messages (LinkedIn and Messenger) sent directly to the participant by the lead researcher. The participants were then contacted by the lead researcher to arrange a time and place for an interview. They were recruited in such a way that their anonymity and personal privacy were ensured.

### Data Collection

This study was carried out in accordance with the guidelines of The Data Protection Official for Research at NSD, The Norwegian Center for Research Data AS. All participants provided their written informed consent prior to the interview.

Semi-structured, face-to-face, one-on-one interviews were conducted with all participants, to retrieve information and to elicit experiences, thoughts, feelings, values and opinions (Joffe, 2012). Qualitative interviews were considered to be the most suitable data collection method for completing the core set of open-ended questions, and for allowing the flexibility and flow of additional questions to be attuned to the dialogue of the interview (Patton, 2002; Brinkmann and Kvale, 2015). This approach to elite interviews with professional experts furthermore seeks to entail mutuality, relevance and to increase understanding (Brinkmann and Kvale, 2005). Semi-structured interviews also gave the participant the opportunity to bring up new themes and issues (Hennink et al., 2011).

The first author was the sole interviewer in this study. The second author, however, supervised the interview process closely with regard to information, the interview protocol, facilitation of the interview situation, and a summary of impressions after each interview. Information on the research project and a definition of the unforeseen was provided to interviewees in the introduction section of the interview, to ensure a common frame of reference. The participants were asked, at the beginning of the interview, to tell their career story in a way that was meaningful to them. The pre-structured open-ended questions included in the interview protocol were derived from previous research and current literature on the unforeseen (see e.g., Herberg et al., 2018, 2019; Torgersen, 2018a). The length of interviews and order of questions varied. All participants were, however, asked questions from six broad categories; (1) experiences and lessons learned from unforeseen events, e.g., *What are the most important things you have learned from these experiences?* (2) the importance of differences between individuals, e.g., *How have differences between individuals been reflected in the unforeseen events you have experienced?* (3) the importance of social factors, e.g., *What keeps the team/group together when something surprising, unknown, and unlikely happens?* (4) the importance of educational structures, e.g., *How do you consider the ability to carry out concurrent learning?* (5) the importance of organizational factors, e.g., *How do you stimulate people to master when under risk, when something is unknown and where the chance that something will go wrong is present?* (6) the importance of operational factors, e.g., *How do you experience the relationship between planning, preparing and handling an event?* Probing techniques were used to ensure more detailed explanations, data richness and quality (Creswell and Creswell, 2018). It was ended with a summary, the interview at this stage opening up for clarifying questions and final reflections.

The 13 interviews lasted an average of 90 min (range 49 min to 118 min). This gave 1104 min of interview data (18.4 h). Nine interviews took place at a discreet location in an office outside the workplace of the interviewer and the interviewees. Three interviews were conducted at the participants’ workplace. One interview took place in a hotel room. The interviews were digitally recorded and subsequently (a) transformed to computer audio files, (b) anonymized, (c) secured with a password, (d) deleted from the digital recorder, and (e) professionally transcribed verbatim before analysis. All of the participants were given the opportunity to view and review the transcripts. The transcriptions yielded 350 single-spaced pages of text and 148,258 words.

### Data Analysis

Thematic Analysis (Braun and Clarke, 2006, 2013; Clarke and Braun, 2013) was applied to the qualitative data collected from the participant interviews. This method gave a flexible approach to the semi-structured interview data. It also provided a framework and a consistency and coherence when identifying patterns, and when developing meanings and themes within the dataset (Braun and Clarke, 2006; Nowell et al., 2017). The themes were patterns of explicit (manifest) and implicit (latent) content that relate to issues such as *individual characteristics and competencies* and *the role of emotions* in coping with unforeseen events (Joffe, 2012, p. 209). The interview protocol and the research question gave some predetermined categories for data analysis examination. This study therefore primarily draws on the specific form of a top-down theoretical thematic analysis (Percy et al., 2015). The analysis was, however, also partly driven by the data itself in a bottom-up or inductive process (Braun and Clarke, 2006). A dual deductive/inductive and latent/manifest set of themes were therefore employed together (Joffe, 2012, p. 210). The qualitative data analysis software NVivo (version 12.5.0 Mac) was used to carry out the analysis, which was based on the six-step procedure and similar to that proposed by Braun and Clarke (2006, 2013). The analysis advanced from one step to the next. The steps were, however, not necessarily linear, but moved forward and backward.

The transcripts were read several times in the familiarization stage. The audio file of interviews with each participant were also listened to. Words, metaphors, sentences, and phrases that appeared intuitively meaningful were, at this stage, written down in a memo before advancing to the next stage (Percy et al., 2015).

**FIGURE 1:**
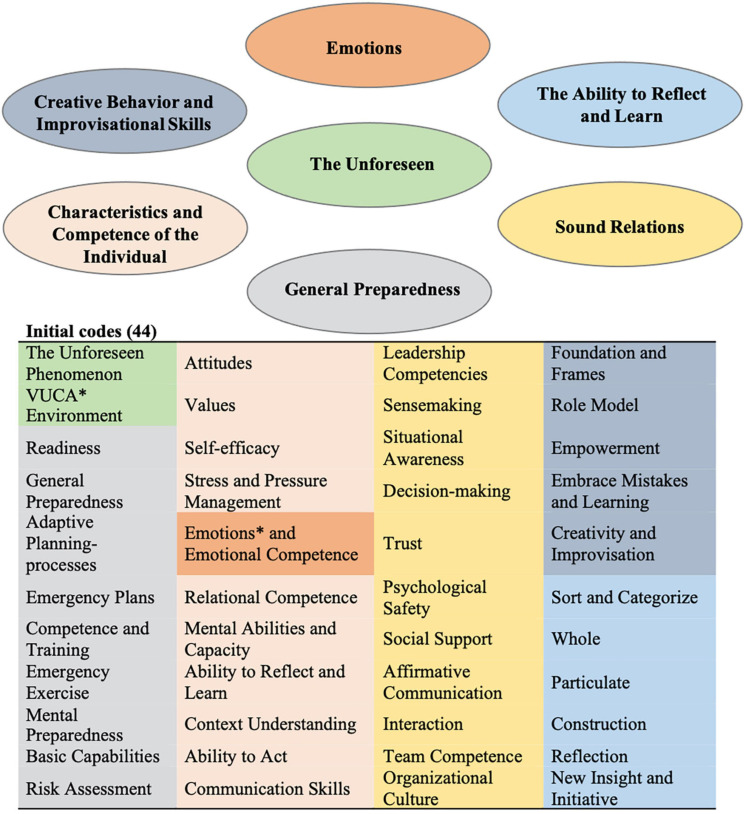
Map of overall themes derived from initial coding. The colored background represents the linking and grouping of the initial codes into emerging overall themes. ^∗^VUCA, Volatile, Uncertain, Complex, and Ambiguous (Barber, 1992). ^∗^Emotions emerged as a main theme evident across all professional experts. Initial sub-coding of emotions: negative emotions, positive emotions, fear, shock, panic, surprise, interest, mood, language, metaphors, metaphorical expressions, sensemaking, levels of emotions, emotional capital, emotional intelligence, emotional dissonance, emotional incongruence, emotionally contagious.

The transcripts were then thoroughly and systematically explored, each data item being given full and equal attention to generate initial codes (Nowell et al., 2017). Segments of data in each transcript that seemed relevant or that captured something that was of interest to the research question, were provisionally coded. New codes were generated. Existing codes were sometimes modified as the analysis progressed (Maguire and Delahunt, 2017). Memos were recorded to identify, reflect upon and discuss interesting aspects and ideas around codes. The initial 44 codes are shown in Figure 1.

The third step centered on the search and generation of higher-level themes and subthemes, in an iterative process based on the coded data. For example, there were several codes that related to *interaction, relations* and *emotions*. This study covered a wide variety of concepts, and the pre-determined deductive codes from the interview protocol therefore helped organize the data into main themes. Most of the sub-themes were, however, developed inductively. The themes were predominately descriptive, but also interpretive. Figure 1 shows a thematic map which we used to assist our organization and link the initial codes into broader themes (Braun and Clarke, 2006).

The preliminary themes from step three were then reviewed, modified and developed. The coded data for the sub-themes was reviewed to determine whether there was a coherent pattern that was relevant to each theme in the context of the entire data set (Nowell et al., 2017). The second author reviewed some of the first author’s interviews, transcripts and coding. The second author also engaged in co-analysis of theory and previous findings. Some single themes and sub-themes were eliminated, collapsed, and new themes and sub-themes were created.

**FIGURE 2:**
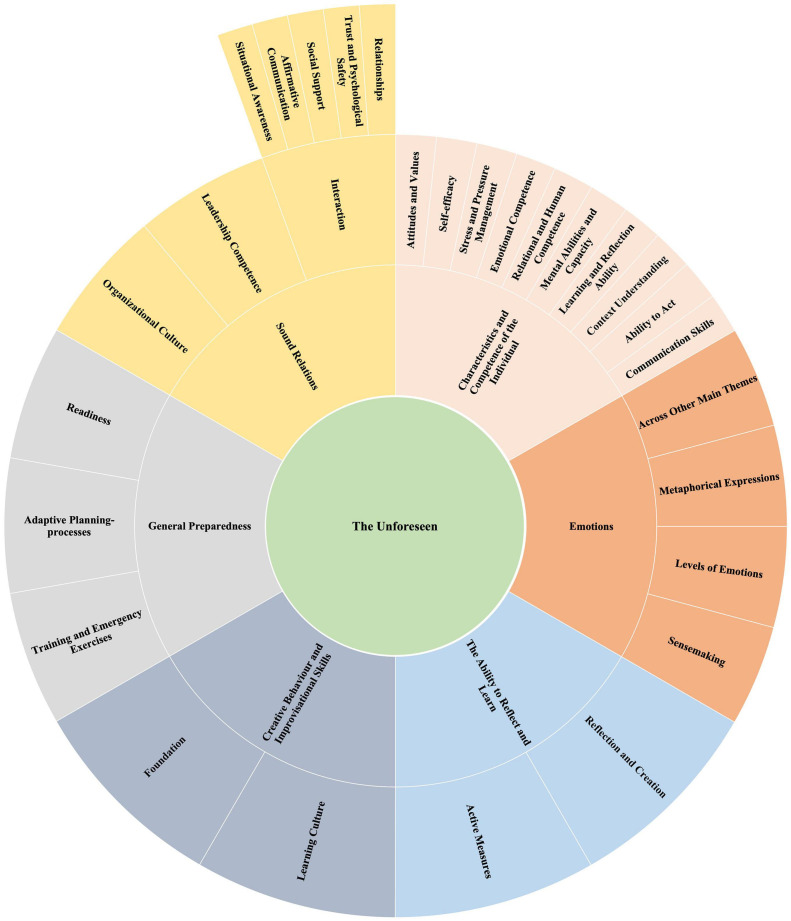
Sunburst map of the seven main themes, including sub-themes and sub-sub-themes. The map also represents a panarchy of three adaptive cross-level cycles (Holling and Gunderson, 2002) based on the main types of competence, units of competence, and elements of competence (Burke, 1990; Truelove, 1995).

A final refinement and detailed analysis of each theme was then conducted, the aim being to “…identify the ‘essence’ of what each theme was about” (Braun and Clarke, 2006, p. 92). How a theme fits into the overall story of the entire data set, and how it relates to the research question, was also considered (Nowell et al., 2017, p. 10). Figure 2 shows a final thematic map of the seven themes etc., these being ordered in a way that best reflects the data.

The analysis was, in this the last and sixth step, finalized once the final themes were established. All the themes were discussed during the writing of the manuscript, to theorize the significance of the patterns and their broader meanings and implications in relation to the literature (Braun and Clarke, 2006) and praxis. Shorter quotes and longer block quotes that best represent the research findings and relevance were selected from the interview transcripts, including quotes from all participants (Nowell et al., 2017).

## Findings

The results of the analysis and the interpretation of the interview data are presented as key themes and are related to each of the two research questions that guided data analysis. The unforeseen was first explored based on participant experience, potential types of resilience competence then being examined based on the interview guide. The role of emotions was, however, uncovered by the inductive data analysis process. The presence and impact of emotions in response to unforeseen events was evident to all professional experts, so emerging as the main theme. Seven broad thematic groupings emerged from the analysis of the data, as shown in Figures 1, 2.

### The Unforeseen

*The Unforeseen* emerged as the first rich and complex theme participants highlighted from their experiences and reflections, as shown in Table 3. This was somewhat expected, as all study participants were selected because of their unique and extensive knowledge of dealing with some of the most challenging and unforeseen events that have affected Norway in recent times, including the terrorist attack of 22 July 2011. Three separate sub-themes materialized from the data. These were *The Unforeseen Phenomenon, The Experience of an Unforeseen Event*, and *Temporary Shortcomings.*

**TABLE 3:** Quotes from experts illustrating experience with the unforeseen as a phenomenon.

**Theme**	**Illustrative quotes**
The Unforeseen	…It wasn’t something that we had planned for, no one saw it coming, and it [the event] hit pretty fast. (P5)
	It came out of the blue, it was not the sort of event you go and prepare for …So, we were completely unprepared for it, it came like lightning from a clear blue sky. (P12)
	…events like these are so dramatic, they come like lightning from a clear blue sky, even though they are within the bounds of what you might expect. (P7)
	We were very unprepared for it, never thought about it happening. (P2)
	…it was one of those blindside events, absolutely no warning. (P8)
	…it turned everything I had thought through upside down. (P9)
	There was no plan for that scenario, obviously a gray zone, it was a black [swan], we didn’t see it coming. (P4)
	Unforeseen, but it’s difficult to say that it was totally unforeseen. You may have thought about it happening, but it’s not something we’re trained for, there was something unforeseen at the heart of it. (P13)
	…it’s pretty obfuscated, but it became a quite special event, because we got so many attack points that I don’t have a record of …And then it starts to get a little unforeseen, because I wasn’t quite prepared for it, and then it starts to get difficult. (P10)
	…everything was rational, all the plans were prepared, everything made sense when pinned up on the wall, the strategy, and all that …(P11)
	You can never really prepare 100% for lightning from a clear blue sky. You just can’t. That’s precisely the point. What happens is something you’ve never thought about at all. (P12)
	I don’t think you can prepare for what’s coming, but you have to be prepared for it, at all levels, even in your private life. (P9)
	The slightly strange combination of completely unforeseen, that’s how it was going to happen, but foreseen in that the terrorist threat and risk had been raised. (P6).
	A lot of people were in complete shock when it happened, but it was one of the scenarios we had been discussing for a long time. (P1)
	Accepting that things do not go as planned, I think that’s a challenge, particularly perhaps for us in Norway with such a trust-based society. (P7)
	No matter how much you plan and prepare a black swan can always crop up…, and then you just have to handle it to the best of your ability. (P13)
	We have the knowledge in the unit, we have what it takes to solve the unforeseen. The unforeseen, it always comes, but you just don’t know when. (P3)
	The vast majority of the unforeseen events I have been involved in, through my role and function, have very often required some form of action. (P6)

*The Unforeseen Phenomenon* is the first sub-theme the participants reflected on. One of the participants captured some of the complexity of defining an event as unforeseen:

*We didn’t see that one coming, really. But at the same time, it was conceivable, of what a threat actor in the area is capable of, in theory. So, it was not totally out of the blue* (P4).

Some of the quotes in Table 3 also shed light on different aspects of the phenomenon. Many use metaphors to describe the phenomenon such as; *lightning from a clear blue sky, out of the blue, black swan*. An imaginary, subjective and objective dimension is created based on probability, predictability and on a degree of familiarity with operating in a continuum in the period of time after the event takes place. There may also be unforeseen elements at different points in time within the event itself, as participant 3 points out:

*One talks about unforeseen events, I think most of the events we have, they are unforeseen …they always have something unique and new about them, …* (P3).

Perspective is a key aspect. What is unforeseen for one person, group, or organization is not necessarily unforeseen for others. Perception, understanding, and context therefore play a real and practical role when an incident occurs that needs to be handled:

… *it was thought of, scenarios for it were made, assessments were made around it…, but no one foresaw it completely anyway* (P6).

All of the participants described *The Experience of an Unforeseen Event*. The events are often described as being; *complex, dramatic, demanding* and *unclear*. They happen quickly and without any registered or identified warnings. Warning signs may, however, have been overlooked or may not have been taken seriously. This often makes the experience overwhelming and chaotic. Reflection in the aftermath of the incident, or when things become more normalized, indicate that the event was *unexpected, unlikely, unthought of, unplanned, not prepared and not trained for*. It is reasonable to assume that the subjective experience arises from roles and functions, and from what was prepared, trained and practiced for prior to the event. The experience of the event is therefore a function of anticipations and preparation.

*Temporary Shortcomings* is a type of experience that has been grouped and coded as the third sub-theme. The participants point out that there is often, in an unforeseen event, a sense of individual or collective shortcoming that at worst can temporarily shock and paralyze. The urgency to act at the same time encroaches. All study participants therefore stressed that it is crucial to move from a reaction mode, to a mode in which one is able to act quickly and appropriate. Competence and coping skills are quickly assessed unconsciously and deliberately in relation to the situation. If the gap between competence possessed and that required is perceived to be large, and that the event *lies on the fringes of what anyone had imagined, …then crisis management essentially becomes a question of how well prepared one is* (P6). There is often, however, a recognition that the measures and barriers set up to prevent a threat have not worked as planned. The majority of participants therefore report that the primary objective becomes managing the event or the situation to the best of their ability.

As such, the findings, that relate to the first research question of this study, show that the unforeseen is a complex phenomenon, the potential to surprise and at worst shock people and organizations depending on perspective and subjective experience. The professional experts’ experiences show that the ability to respond, adapt and change must match the nature of the event occurring to be successful.

### Competence for the Unforeseen

#### General Preparedness

The second theme (main type of competence) that emerged was the importance of *General Preparedness*. The quotes in Table 4 show the span of general preparedness to be wide. Plans, practice, training, structure, and equipment are often mentioned as important elements. There are, based on the initial coding, however three sub-themes (units of competence) of this major theme that stand out from the experiences of the experts. These are; *Readiness, Adaptive Planning-processes, and Training and Emergency Exercise*.

**TABLE 4:** Quotes from experts illustrating five themes related to competence for the unforeseen.

**Themes**	**Illustrative quotes**
General Preparedness	…the more we train and practice specific things, the more locked into them we become. So, if you suddenly have to try something completely different, then recalibrating is very difficult. (P10)
	I think we occasionally get lost in plans based on too many assumptions (P7)
	If you don’t have one of the main elements; you haven’t trained, the plan is not known, or you don’t have the equipment, then it’s game over. Then it gets difficult. (P4)
	It is not insurmountable for most [organizations] to manage to create a good foundation. The next steps from this are, however, a greater challenge. (P5)
Characteristics and Competence of the Individual	Pick people with good attitudes that you can trust, who are empathetic and actually look after the people around them, as I think this makes a group much more robust. (P8)
	There is a type of behavior and character that acts instead of just reacts. (P12)
	…individualists who work in teams and who don’t give up. (P7)
	You need the complete range of competencies to be able to build better answers, or better strategies for handling what happens in the future. (P6)
Sound Relations	I think the ability to be well known across society is something that is important at the individual, group, and especially at the system and structural level, if you are going to solve a problem in which a lot of things are happening at the same time, the situational picture is a little blurry, and it’s a little unclear what you should do. (P6)
	I think you often become, … you get a little narrow minded when the crisis or the unforeseen happens. (P10)
	I’m committed to building that resilience in people, teams and units. But can I do it? Yes, we have good relationships, good values and intent-based leadership. (P3)
	Resources are not unlimited, i.e., personnel. We have to take care of the ones we have. (P8)
Creative Behavior and Improvisational Skills	I also strongly believe you have to have the ability to chuck everything away and think completely freely and new …Improvisation is important. I would say that it’s very important. (P2)
	…to improvise, you need something to improvise with. You must have some tools. You can’t improvise out of nowhere…Flexibility of the mind is one thing, another is flexibility in tool use. (P12)
	You have a deep knowledge of your own capacities…I think it’s about confidence in understanding who you are, where you are, and what you know. Then it’s easier to improvise. How secure are you then in the situation…(P4)
	…creating an opportunity to fail, i.e., glorify the epic failure. I mean, embrace failure, but with learning. (P11)
The Ability to Reflect and Learn	We’re not very good at taking the time for concurrent learning. It’s more that we keep on going until we crash, learn from it, then do something else next time. That’s the short version – concurrent learning is obviously something we don’t do much of. (P12)
	The thing about learning as you go along, is that it ends again with the ability to think critically, the ability to think, that things don’t go as planned. (P7)
	…in the most stressful [situation], then stop for a second, don’t be just driven along by everyone around you. (P9)
	I think that the reflection room…a lot going on demands a lot from the leader, and I think you have to point out that this is essentially a managerial responsibility, and again, take a step back, reflect, and ask a critical question. (P6)

The first sub-theme, *Readiness*, relates to systematics, which is an established structure and organizational culture of people who recognize and accept that small and large unforeseen events will occur no matter how good risk analysis and plans are. One must be prepared to accept that *things do not go as planned* (P7), have *the expertise to cope with a VUCA environment* (P11), *ask questions that can lead us forward* (P6), and *dare to think of the most unthinkable and most challenging situations* (P1).

The second sub-theme highlighted by the professional experts is the importance of flexible and sincere *Adaptive Planning-processes*, that develop applicable emergency plans that are not based on too many assumptions. Risk assessments and equipment must also be available, and personnel must be able to use it. General preparedness is a continuous process and is a foundation that *is not an insurmountable obstacle for* most organizations to establish …(P5). On the other hand; *the next thing that hits will be different to the last* …, *the challenge is the next steps* (P6).

The third sub-theme emphasized by all respondents was that emergency plans and possible scenarios need to be *trained and exercised*. Plans should be trained, and organizations should simulate and practice for a range of situations based on complex and realistic scenarios. *There is a link between what one does day-to-day and what happens in a crisis* (P7). The respondents highlight function and role training, but also practicing personnel ability to change and take on different roles. The focus in emergency exercises should not be too one-sided or scenario specific. One should, preferably, train and exercise with other actors, *practice letting others in*, and *practice conflicts of values and dilemmas* (P6). Some respondents pointed out that this is particularly important in the face of new transboundary threats.

#### Characteristics and Competence of the Individual

The third emergent theme was *Characteristics and Competence of the Individual*. This is a comprehensive theme and involves a number of sub-themes, as shown in Table 5. The purpose of this study is not to delve into all these differences. Three aspects can, however, be used to illuminate the results of the data analysis. First, all experts highlight the experience that different people deal with an unforeseen event in different ways. Respondents point out that this is often desirable, as there is a need for people with a range of qualities in the different phases of an event. Both those *who run fast and those who move slowly* (P6), *the intuitive and analytical* (P2), and *the operational, the strategist and the visionary* (P11) are all needed. The foundation must, however, be competence in their field. Second, a number of the experts also mentioned that it is still not always easy to predict how the individual will react and act, despite good selection and training. This leads to the third aspect which all experts highlight. This is that the individual is to a significant extent affected by the context and the other people they operate with in an event.

**TABLE 5:** Communalities of characteristics and competence of the individual.

Sub-theme*	**Description**
(1) Attitudes and Values	People who are trusting, who take care of others, are accessible and empathetic. Have integrity, are responsible, grateful, humble, and who yield to and believe in the philosophy of leadership and the purpose and values of the organization.
(2) Self-Efficacy	People who believe in themselves, who are self-confident and assertive, but who balance this with humility, doubt and curiosity, who can lead themselves, dare to make mistakes and are ready to make a difference.
(3) Stress and Pressure Management	People who are robust, enduring, secure, calm, pragmatic, rational, sober, thorough, capable and conscientious, can endure a lot, know their own limits, and show the courage required.
(4) Emotional Competence	People who know their own feelings, know and understand the feelings of others, and who can accommodate, regulate, master and show emotions, are emotionally stable and appear secure and present, but have the ability to talk about emotions and the demanding and are able to react and let off steam in an appropriate way.
(5) Relational and Human Competence	People who sense signals, see people, provide support and care, take care of themselves and others, create trust, are inclusive, and who can fit into and collaborate with others.
(6) Mental Abilities and Capacity	People who are mentally prepared, have mental capacity, who can process a lot of information, “raise their gaze,” assess a situation, capture the potential, assess risk, be able to think outside the box, be creative, inventive, and be able to improvise.
(7) Reflection and Learning Ability	People who understand the environment and the situation, and who are able to stop, reflect, adjust, realign, renew and embrace something new and unknown.
(8) Comprehensive Context Understanding	People who see wholeness and relationships, act as a link and as a liaison, who can interact, share information, and can communicate vertically and horizontally within and between organizations.
(9) Ability to Act	People who act, create energy and movement, and who are robust enough to make decisions on limited information during risk events.
(10) Communication Skills	People who share and can articulate themselves and who speak up.

*Units of competence as dimensions of characteristics and competence of the individual (main type of competence).

There are, however, some commonalities across the experiences of the experts interviewed, which characterize those they believe manage unforeseen events better than others. These characteristics and competencies are briefly summarized in the 10 sub-themes in Table 5.

#### Sound Relations

The fourth theme that emerged was the significance of *Sound Relations*. This theme has a coverage that is wide and relates to human interaction at the inter-personal, group and organizational level. The 13 expert interviews provided significant data in this area. The three sub-themes that underlie this key theme were *Interaction, Leadership Competencies*, and *Organizational Culture*.

All participants describe *Interaction* as being a significant relational unit of competence for dealing with unforeseen events. The need to be able to interact increases, particularly when *things move fast, and a lot of information, and decisions are needed* (P3). The five sub-themes of *Interaction* are presented in Table 6. These are elements of competence that the interviewed experts claim promote interaction.

**TABLE 6:** The five sub-themes (elements of competence) of interaction.

Sub-sub theme*	**Description based on participants quotes**
(1) Relationships	*Relationships mean a lot* (P2). *At the management level, it’s really important…, to have personal knowledge of those who you support and whom you interact with* (P7). *A feeling of* s*ecurity is paramount, …build good teams that are confident in each other* (P8), *and quickly create a common mental model* (P13).
(2) Trust	*It’s about trust* (P4), and you *have to declare that you trust, talk about it, and show it* (P7). Build trust *among people who are different, who know different things, have different strengths, and who react differently* (P11). *Trust is the foundation…, by getting to know each other’s strengths and weaknesses, …share experiences and reaction patterns* (P12). *Trust that you and the others are at the expected professional level* (P1), and…*trust in each other as people* (P2). *Including others into decisions builds trust, …seek support and involvement with those with you* (P3).
(3) Social Support	*Genuine social support comes naturally* (P2) and *appears in practice* (P3). *It may support people and help them on to the right path where there is uncertainty on the implementation of measures* (P10). *Support for decisions, planning structure and mandate, must be given in advance, and must be able to function in practice, …full support, including from senior management. How can we support? How can I be the tool that you can use to best deal with the crisis?* (P5). Another aspect of social support is *knowing that you can get emotional support…look people in the eyes with respect and responsibility, …keep a long horizon on caretaking* (P8). Participant 10 believed that *the need for social support and feedback is individual, …but important for motivation and self-confidence*. Participant 13 expressed a *greater need for moral support and feedback after events*. Some of the participants also believed systematic defusing, debrief and colleague/leadership support to be important, and support from specialists where required.
(4) Affirmative Communication	*Affirmative communication* including strategic communication, affecting, emotional and rational, is mentioned by a number of participants as a being a key element in being able to quickly provide a response, convey concrete information, create understanding, give meaning, encourage calmness, make space, remedy pain, and prevent disunity and uncertainty. *When more and more people have something to say, they start to reduce the space for decision-making and space for maneuver. Decisions evaporate* (P3). Participant 6 points out that one is *too weak in the event of large sector-wide threats*. *We need to learn how to deal with new people and new organizations in new situations* (P9). Participant 3 noted that *if you’re going to interact well, you must express when you’re not sure…you have to open up even more with people you don’t know*.
(5) Situational Awareness	*Having good situational awareness* (P6), *establishing a common purpose* (P4), *getting to know cooperative actors, being able to scale up from a foundation, build a structure, and reinforce the team* (P5), *share and use information* (P6), *see links between single events* (P2), *plan for the ultimate potential of the event* (P10), and *clarify the leadership and roles of actors* (P3).

*Elements of competence as dimensions of Interaction (unit of competence) and sound relations (main type of competence).

Participant 12 noted that *the higher up the organization, the weaker the interaction.* Silo-based and hierarchical systems, self-centeredness, unresolved responsibilities, prestige and status, competence disputes and subtle ways of maintaining power were highlighted as elements that inhibit efficient interaction. One must therefore be *good at working across and building relationships* (P7), *at formalized collaboration and co-training with other actors* (P13).

The second sub-theme, *Leadership Competencies*, primarily relates to relational aspects, and to the responsibility of ensuring that people are mentally, emotionally and practically prepared. It also refers to the ability to build situational awareness, and then make relevant decisions. *Leadership is essential, at every level* (P12), the role of leadership being *to maintain the overview, see the whole, use resources, and capacity* (P4). Participant 6 also noted that we need *leaders who take a step back, reflect, ask questions including critical questions*. All participants emphasized the importance of creating good relationships that are based on psychological safety, well-being, openness, recognition, humility, trust and support. Some participants also found that *value-oriented leadership is quick and efficient* (P5), when decisions can be filtered through these values.

Most respondents were, thirdly, concerned with developing an *Organizational Culture* in which there is room for *making decisions based on the information they have* (P5). There is also a need for an acceptance in an organization for *things not going as planned* (P7). You therefore need *values that are deeply impregnated into the organization and that new people are exposed to [these] from day one* (P1). *A culture of reporting what management needs to know, and not of leaders controlling what they think they should know* (P7). Participant 10 also pointed to a familiar challenge, this being that *those out there need to understand what kind of information goes up the system, and those further up need to understand what needs to go out*. All participants express that one must cultivate a culture which is as open and transparent as possible, and where understanding and acceptance is fostered, so that all can share and learn from mistakes. *The person sitting at the top must set the standard* (P2).

#### Creative Behavior and Improvisational Skills

The fifth theme, *Creative Behavior and Improvisational Skills* revolves around *using things in a different way to solve a new problem* (P12). The experts furthermore believe improvisation is expressed at the individual, group, and organizational level. Improvising is having *the skill to develop an organization that is adapted to the event* (P5).

This theme has two sub-themes; *Foundation* and *Learning-oriented Culture*. First, all the experts emphasized that a basic platform is required for improvising under risk. Organizational elements such as overall plans, planning processes, and having useful structures and systems could be important parts of a *Foundation*. *You have to have a foundation or you’re pretty bad at building* (P5). In some cases, it can also involve taking risks, and *breaking established rules and routines* (P8), and *daring to “fuck up”* (P1). Others were, however, clear about that there are *some frames you can’t deviate from, …and not improvise beyond absolute minimum standards in the organization* (P4) or *aggravate the situation* (P13).

Second, in building a *Learning Culture*, many experts highlight the contradiction between nurturing self-efficacy and the risk of making mistakes. *It’s hard, because nobody wants to fail* (P11). Several experts point out the importance of increasing the ability to improvise and of *building knowledge, expertise and security step by step, complexity increasing. You then create self-efficacy. That’s the key* (P5). *Add new elements when practicing* (P10). *As leaders, present good examples, …preferably the younger* (P3). *Share and tell stories about things that were done in a different way* (P9). *When you, as a leader, know what needs to be achieved, then you can improvise to a greater extent than you can when you just follow a recipe* (P4). Intent-based leadership therefore creates room to improvise.

#### The Ability to Reflect and Learn

The sixth theme, *The Ability to Reflect and Learn*, consists of the two sub-themes *Reflection and Creation* and *Active Measures* to exploit learning opportunities in an ongoing event, both individually and together with others.

*Procedures are good when things move fast, but sometimes opportunities open up …and it’s important to see them …especially when procedures no longer can help you* (P1).

One must first create room for *Reflection and Creation*, take a step back, pause for a moment. Then you can get an overview and start asking the critical and the right questions. Share with others, *close-loop communication* (P3), and, if possible, *challenge, ask and discuss* (P7). Then you can construct by *putting little things together into a bigger picture* (P13), through focusing on capturing signals and details, active listening, sorting and categorization, and by using all the senses and elements from past experiences.

Several participants, however, pointed out that it is difficult and demanding to change perspective and learn during an extreme event, where things are stressful and moving fast. Others said that concurrent learning was something new, something they don’t train for, or don’t have a system for. *Drill, plan and decision – always, …concurrent learning, not something I place much pride in, …haven’t seen much of it. Pit stops and reflections, not much of that either*. *Our way is very extrovert and characterized by action …* (P12).

There is *often an inherent need for action, without asking whether it is relevant, …action will quickly overcome the ability to stop* (P1). Some participants therefore pointed out that *Active Measures* must be taken, and that this is a managerial responsibility. *I believe that without deliberately taking steps to learn concurrently, then it just won’t happen* (P4). Perhaps it is also *easier for a leader than those who are directly involved* (P10), and that one probably has *greater capability at the strategic level to stop and think through, than in an operative situation* (P6). *Do we reach our goal? Does the plan work? Does the organization meet the need?* (P10). The changes that should be made are not necessarily large, …*they don’t have to be big, but tricks or adjustments may make you handle the event a notch better* (P5).

In sum, the findings of research question two show that the five main themes or types of competence, their units and elements, nuance and reinforce previous research. Furthermore, they provide a richer and deeper understanding of the concepts, factors, and inter-relations between them. The findings also suggest the practical importance of these to those who manage unforeseen events.

### Emotions

Emotions emerged as a consistent and central seventh theme, explicitly and latently across all participants in the study, emerging primarily from the inductive analysis. Emotions were firstly related to all the other main themes. The role and meaning of emotions were also illuminated through the language and metaphorical expressions the participants used to describe their experiences. Thirdly emotions, through the analysis of the reflections and examples the participants highlighted, appeared at different levels, spanning from the inner individual to the institutional level. Finally, emotions also seem to play an important role in determining how a situation is perceived and understood, in creating meaning and self-efficacy, and in creating the basis for required actions and decisions.

#### Emotions Across the Other Main Types of Competence

Table 7 shows the role and significance of emotions in the other five main themes were expressed in a number of ways in the participants’ descriptions and reflections.

**TABLE 7:** Quotes illustrating the role of emotions across the other main types of competence.

**Themes**	**Illustrative quotes**
General Preparedness	Lightning from a clear blue sky …the moment of surprise, is so massive, I think it takes longer to sort out the toolbox you have, before you can start dealing with it.
	…first of all, you have an emotional reaction, that this is something that is created because things have not gone as planned. (P11)
	You can get used to anything that is uncomfortable if you are exposed to it in a sensible, safe frame, then you will get better and better. (P7)
Characteristics and Competence of the Individual	There are some who have their strength in maintaining an outward calm, making good decisions and continuing to play the ball even when things start getting crazy. Then there are others who enter a mode in which they just make decisions and keep on pushing forward. (P1)
	People have fallen out, simply haven’t functioned, where they maybe have been paralyzed by fear and haven’t functioned rationally. (P8)
	Being able to relate to others within what we call the EQ-domains, i.e., being able to see people, pick up on signals, weak signals, recognize early the indicators that things are about to go well or bad, I think are important. (P4)
Sound Relations	…it’s, I think, a lot about actually knowing some of the range of the feelings of the others. Becoming more confident in those that we see have contact with themselves, but at the same time that we can perform together, that there is some control of it [the feelings] when we are to achieve something. (P9)
	…knowing that you have people around you, that there is a security in being able to say; “now I’m scared,” and in being honest. (P8)
	…this is about emotional capital. The capital you build on, which is then purpose driven. I have an unceasing belief that having a strong purpose makes it easier to deal with what’s coming, and interact. (P11)
Creative Behavior and Improvisational Skills	Is there a difference between those who handle it well and those who don’t? I’m talking about things like emotional stability, the ability to use what you know. It’s improvisation in practice. (P12)
	You never experience that an incident, or the handling of an incident goes by the book…you always have to improvise – no matter what, you have to improvise. But knowing in advance that it is expected of you, and being aware that this is the nature of the event, also provides a reassurance. (P2)
	I don’t think you should be afraid of making mistakes. I think I’ve been a little too afraid to make mistakes, been a little too…wait, I’m a little too “blue” [conservative], so I’m a little …“No, we’re not doing this.” (P6)
The Ability to Reflect and Learn	…especially when we start to get into some very demanding situations. What happened? …now we have to gather ourselves, OK? But you very often don’t have time for this in the most stressful situations, so to learn in the moment, it’s very demanding. (P9)
	Especially if non-traditional events occur, or they come suddenly, or they have a complexity you’re not used to, then you might take a familiar track or get a little stuck. (P5)
	I have the ten second rule. Active listening, which gets easier the older you get, but also being able to stop yourself emotionally reacting straight away, to take 10 s to reflect on the situation, …wait and take a moment of quiet. (P11)

*General Preparedness* relates to being prepared for things not going as planned. It is impossible to be completely prepared. *We’re not going to be prepared for the next event either* (P7). There will therefore always be an element of confusion, doubt, surprise and an emotional reaction when something unforeseen happens. Several participants highlighted that the development of knowledge and skills in managing their own and others’ emotions and reaction patterns is important. Participant 13, however, addressed that emotions can also play a role that is challenging:

*You don’t want to step outside your comfort zone and train for something that’s unknown.*

Another aspect of emotions relates to *Characteristics and Competence of the Individual*:

*It is very interesting to see that some have a much more developed ability to talk about emotions and demanding experiences, when they are down and exhausted. We often think of these people as being the toughest of the tough* (P9).

The quotes in Table 7 furthermore show the difference between how people connect with, accommodate, show, and use emotions. The ability of some people to capture and understand the emotions of others, emotional stability, and level of experience of stressful and demanding situations also varies.

Another comprehensive main theme in which emotions are emphasized as an important element, is *Sound Relations*. This is mainly expressed in relations between individuals, in groups, in leadership, and in the organizational climate. A number of participants underlined the importance of good relationships, of knowing each other personally, of creating shared experiences of stress and pressure, and of knowing how others react. All participants furthermore emphasized the importance of leaders establishing such relations and a social climate:

*I think you have to learn to balance emotions …, especially as a leader, because leaders need to think that we don’t just need to deal with our own (emotions), but we also have to appear to be so confident that the people going with us are confident that we’re going in the right direction* (P9).

Emotions also appear to influence *Creative Behavior and Improvisational Skills*. The vast majority of participants highlighted the importance of creating a working environment in which the fear of making mistakes is not too significant, as this inhibits the ability to improvise. This is particularly true in situations that involve risk, and where the outcome is uncertain. Most of the participants pointed out that experiencing support and emotional safety can, in such situations, contribute to critical thinking and to the arousal of interest and curiosity.

Finally, all participants related emotions to *The Ability to Reflect and Learn*, the inability to master uncertainty, and the discomfort and stress this results in affecting the ability to reflect and learn as situations unfold. Some participants pointed out that it sometimes is easy to *run on autopilot* or to choose solutions that, based on experience, one is familiar with. This pattern can be further reinforced by the time and information pressure in unforeseen events. There is therefore a risk of overlooking important aspects that could lead to other and better courses of action. Several participants emphasized the importance, in such situations, of the ability to stop, communicate and share with those around them:

*If two people face their insecurities, then it is much better that they meet and confirm for each other. I think it would provide a sense of security* (P10).

#### Metaphorical Expressions Related to Emotions

The data analysis next showed that participants often used metaphorical expressions of emotions to describe their experiences with the unforeseen. The quotes in Table 8 give an insight into the function of emotions, types of emotion and intensity, and the components of emotions.

**TABLE 8:** Quotes from experts illustrating metaphorical expressions of emotions.

**Metaphorical expressions**
So that, most important experience, well it’s to *lower your shoulders* a little no matter what *hit you*, if you can. (P6)
Some *go into overload* when things happen, without you having been aware beforehand that they would not be able to handle it…some just *drop completely out*. They *stop functioning completely.* (P4)
Where you see someone *drop completely out due to stress* – some people can handle it better than others. I have not found a model that can show which ones will. (P11)
It almost gave me a *paralyzing fear*, and it *lasted* quite a while. (P8)
Sometimes I say it’s *at the tips of your fingers*, because you have experience and so more quickly recognize how people react physically, so you see it. (P11)
The closer you are to where *lightning strikes*, the greater the chance you are *knocked off balance* and unable to handle it. (P12)
…selection is at the bottom of it all…, which in a way sifts out those that do not meet these character traits or patterns of behavior and means that they will not be able handle even an ordinary crisis. I mean, they panic, *they freeze, they lock up, or run away*. (P12)
Those who are relatively new to the job are…we see that they have what we call *jazz foot and signal shock*. (P13)
Somebody said to me *“You are Dovrefjell”* (a mountain). “You’re the one it’s good to talk to, you land things.” And that reassurance…if you manage to achieve such a sense of security and responsibility in a group, then I think you have come a very long way. You teach people not to think “I,” but “we.” I think there is a lot in that. (P8)
…when you know you can lean back, because back there are *two sturdy fists that watch your back*, and which you know are watching out for you. (P8)
… to build a culture in which you have experienced you have support. Yes, support in failing, I see that. But in general, being yourself, all you are, and building the trust it then provides, as I think that makes people dare to try more, dare to face the unforeseen, *without going into lockdown*. (P9)
When you’re outside …, it’s fine to be *outside the box*, but when you take it too far, or *don’t have what we call game face* at all. Then, you’ll be told, pretty clearly too, what’s okay and what’s not okay. (P13)
If I don’t have an overview and it’s urgent, then I send out those with the *chaos pilot* quality, to put it like that. (P2)
…I knew I *wasn’t freaking out*, that I was *going to black out*, or I had to *sit down*. I knew I was handling it…and it gave me calm and a feeling of mastery, really. Then I thought “*I’m still functioning.* I’m in control of this.” (P4)
If something happens now that I feel is demanding, then that is because it is very much at the edge of what I work with, or have worked with in the past, where my “*experience backpack*” does not lie. But then you fall back on …, and have faith in, that 80 percent information, and an 80 percent solution is good enough. (P3)

The metaphorical expressions show that the perception and interpretation of an event can bring sensory, bodily, emotional and cognitive experiences together in a way that has an impact on behavior. The expressions also seem to affect the way participants perceive situations and events. The metaphors are strong, functional and illustrative, and perhaps an expression of experiences that are difficult to describe in words. Many of the metaphors simultaneously use a mechanical, practical and rational language to describe what happens to people in unforeseen events; *they go into lockdown and no longer function*.

The participants described negative emotions related to anxiety, fear, surprise and panic, but also positive emotions such as joy, excitement, interest and calmness. A number of the quotes in Table 8 show that many of the participants have experienced that the most appropriate behavior is to try and remain calm, to stand tall, and remain balanced when something unforeseen occurs. For example, *freezing up*, *overload*, and *being knocked off balance* are considered inappropriate, and cause a sense of self-efficacy to fade. This sensory and bodily experience is transferred to an emotional state. The participants therefore perceive the expressions *remain calm, let your shoulders drop*, and *having the mental capacity* as being useful in dealing with something unforeseen. There is therefore a relationship between the intensity and duration of emotions and effective behavior. Most of the metaphorical expressions were used by the participants to describe the behavioral component of emotions. Some expressions also describe bodily reaction. The degree to which the experience component is expressed does, however, vary.

#### Five Levels of Emotions

The expert interview data additionally showed that the role of emotions appeared at different emotional levels. Table 9 shows illustrative quotes from the interviewed experts at five levels (Ashkanasy et al., 2017).

**TABLE 9:** Quotes from experts illustrating five levels of emotions.

**Level of emotion**	**Illustrative quotes**
Within-person variability	…knowing your own feelings will help you a lot in that type of [unforeseen] situation. That you’ve actually felt whether it’s fear or grief and everything you can withstand…, makes it easier in new episodes, because you recognize what’s happening to you. (P9)
	…I felt it coming, and I felt myself opening up, and then I cried properly, I sobbed, and I did it alone and thought that …, now it’s okay, now you can open up. And it was like crying yourself empty, most of all it was a release. (P8)
	I was completely calm, artificially calm, was very switched on, palpitations and the other things you experienced before were gone …, became very focused, as it’s important to be focused and calm. Then my talking became extremely calm, I became very focused on eye contact with those around me…on radiating calmness. (P4)
Between-persons differences	What I have found in stress and in …, when things don’t go right and the model doesn’t work, the people I have, they react to …, they are extremely different, because they have been chosen because they are like that. (P11)
	So, it’s often that there are…that is …, people react in a different way than you thought they would. (P9)
	I have experienced some people becoming very invasive, who may not have been able to keep calm, others become calmer. I think maybe it has at the bottom of it all to do with confidence, which maybe just is there in the individual. (P8)
Inter-personal relationships	There have been cases…where you see that someone is overloaded, and some freeze, then you have to grab them and say, okay, what can we do for you here, now? (P1)
	…really talk about what happened, how did this feel? And how did I act when I felt it. It’s often your inner feelings, but also very much tied to relationships when you’re in situations like this. What happened between us? (P9)
	…I was on the edge of crying, because suddenly there was someone on the other end who actually understood what this was about, and I thought that now I can let my guard down…(P8)
Groups and teams, including leadership	My job as a leader really is to monitor and see that they’re actually hanging in there, and I do that with eye contact, body language, repeating back to me …, what I said to you and everything else verifies that they’ve actually been hanging in there. (P3)
	So, then there is fear, where you are in the management team and how you react is critical, can create a situation where people will flee. They will abandon “ship,” they will not give honest answers, and I remember that it becomes incredibly stressful for me too, because I know that I am responsible for this…when the emotional happens, and decisions are made…The only way I can rein it in, when it gets extreme, is to take them back to why they’re here. (P11)
	The community is created by being exposed to joint pressure and stress, and the feeling of mastery. Being under stress and pressure in practice and training creates a feeling of mastery, and the feeling that we are mastering as a team better than we master individually, is part of the glue. (P12)
Organization as a whole	…there is such an anxiety in other actors about practicing interagency…, I am a little worried that we have created fiercely bureaucratic systems that have cultivated a very strong belief in the system. Loyal leaders who do not dare to come forward and deal with the most demanding events, in which things will go wrong. I’m a little afraid that we’ll get paralyzed leaders who won’t be able to withstand it. (P2)
	No one should be afraid that they will get an assignment in which they think “I hope it doesn’t happen because I’m bad at that.”…try to prepare ourselves as well as possible …, so no one has a bad gut feeling. Then we have to sit down, and we have to train for it, and at least talk about it. (P13)
	So, it becomes important in what the relationships become over time, that you have done or experienced things together that have challenged us emotionally, both for good and bad. And in that way being confident that …, they’re there for you. (P9)

The first level, *within-person*, showed temporal variations in affect and behavior. The participants highlighted, in particular, getting to know their own emotions and reaction patterns. This allowed them to accommodate strong emotions and regulate them in an appropriate way before, during and after events. The second level, *between persons*, shows variability in individual dispositions and characteristics, personalities, trait affectivity and emotional competence. All participants mentioned that people react differently to unforeseen events. The participants, however, emphasized the importance of being emotionally stable, staying calm, managing stress, and feeling confident. The role of emotions in *inter-personal relationships and processes* at the third level shows the importance of interpersonal perceptions and communication, including emotional labor. Several participants underlined the importance of having sound relations. This implies knowing each other personally and sharing and helping each other at an emotional level, as well as at a practical level. Analysis at the fourth level, *groups and team*, shows that emotions can play an important role in team-development and leadership. Several participants stressed the importance of creating an emotionally safe working environment that is based on trust, open and clear communication and a common purpose. Shared experiences in demanding and new situations, can give a sense of self-efficacy at the group level. The role of leader was highlighted by the participants in particular as essential, both as a role model and as a facilitator. The findings finally show the prominence of emotions at the overarching institutional level, at the fifth level *organization-wide*. The affective culture and climate are the organization’s emotional capital. A few participants mentioned emotions at this level directly, as this can mobilize positive emotions, meaning and energy to cope with unforeseen events.

#### Emotions and Cognition

The participants unanimously describe the process of reacting to an unforeseen event as starting with being surprised, experiencing a moment of feeling stunned, or of experiencing a feeling of paralyzing fear, and at worst reacting with shock and panic. Table 10 shows that a common element in participants’ experience is that emotions appear to explicitly influence sensemaking, situational understanding, assessing risk, and the ability to make decisions. Both negative and positive experiences were illuminated in the space of time between the stimulus, the event occurring, and the response.

**TABLE 10:** Quotes from experts illustrating the role of emotions in sensemaking and cognition.

**Theme**	**Illustrative quotes**
Sense-making	But immediately it happens, you don’t really have anything. There and then, in the split second the unforeseen happen, then you only really have the shock, then you have to try to move into a different mode to deal with it. (P12)
	The mental process is important, I think it’s the most important, to understand that you can’t prepare, you don’t know what’s going to happen, I think that’s the most important aspect, to acknowledge this. (P7)
	All people get to a point where they can’t stay calm anymore, and then you can’t think in a structured, systematic and creative way (P9)
	The problem is often that you have an inherent need for action, without really asking the question of whether the action is relevant. (P6)
	What can make you stumble…, or feel like you’re very unprepared? If you get very much locked into your situational awareness. I think there’s a lot in being open to that the real situation is not the way you think it is. (P10)
	I guess I’ve experienced that I become calm when things happen, at least outwardly. I feel the tension internally, but I’ve also been told that I appear calm and that I analyze and look for, “what if”? (P8)
	…if I see people around me who are not in touch with their feelings, and who are generally very secure or radiate self-confidence, then I actually will be in doubt about them when the real unforeseen comes, as I wonder whether they will just go on autopilot for a solution or whether they are as creative as one must be in such situations, to contribute to finding a solution to something completely unforeseen. (P9)
	Big, unforeseen things hit your emotions hard, but that switch is sort of turned off. I noticed that after I had kids, … when I’m at events with kids, then I’m not always so good at controlling my emotions. Thoughts go back to the kids, … it comes before the event is finished. (P10)
	…that you deceive yourself. That’s what you do, you saw …, or see, perceive, decide, act. It wasn’t what you did, you messed with the sequence, or there was something with your ability to see. Your perception was different, and that you have some mechanisms that fool you. (P4)
	If you can manage to liberate mental capacity, then I think it’s easier…I think the more experience you have, the more likely you are to be able to adjust and look for opportunities. (P5)
	The better you know your own feelings, the better you can eventually become at shutting down or regulating. (P9)
	I’m a little too “blue” [conservative], so I’m a little like; “No, we’re not going to do this.” It’s often quite useful…We haven’t set off in the wrong direction. I probably have skills that are weak on action occasionally, so I need sometimes someone to say, “we go now.” (P6)
	…I can start thinking a lot of thoughts right away. So, it becomes very important to find space for the calmness you need to clear your mind quickly. (P9)
	…if you dare to speak up when you are stressed, or the colleague next to you says when he sees you are stressed. The ability to take such a supporting step, as one says; to gather yourself a little. And when you have that information, you’re going to make a decision, and usually it’s more than good enough. (P3)
	…it’s open, it’s transparency, it’s the dialectical process. That they can bring their personal “I” into the team, and not be afraid when they get too emotional or get too …; “Yes, but that’s the way you are, and that’s ok. But let’s move a little out of it.” (P11)
	I think we often start too late to think about the totality and further ahead, as we get stuck in some sort of crisis management instead of lifting our eyes. (P9)
	Threats that you don’t see, i.e., the abstract, become difficult to relate to. And it’s a cognitive challenge for us who try to convince people that the threat is actually real. (P4)

The quotes in Table 10 at the same time show that a number of participants stated that everyone has a tolerance limit, beyond which it is not possible to *think in a structured, systematic and creative way*. Other participants mention that there often is a difference between what a person feels inside and what they show outward. They don’t want to influence the mood negatively by showing negative emotions such as anxiety and fear. This means that many people experience an emotionally incongruent state. There may be an emotional dissonance between what one experiences as a person, as a person in a professional role, and the cultural norms. Emotions are also, in some cases, suppressed. The data showed that some people “*switch off*” their emotions in the situation, others choosing to remain in touch with their emotions, to regulate and use them.

*I have always admired those who appear very secure and calm in situations. I think this creates a sense of emotional safety among people. And I think many of these people are also good at thinking more clearly because they remain calm. However, I have also seen those who are very confident on the outside not acting as rationally as their outside would suggest* (P9).

The analysis showed that emotions are essentially involved in sensemaking in two different ways. Negative emotions are frequently mentioned when routine and the familiar are disrupted by an unforeseen event. Participants pointed out that negative emotions such as fear, anxiety, shock and panic can significantly hinder sensemaking efforts. For example, some of the participants mentioned *that one can become locked within the situational awareness you have, that you run on autopilot, or you do not question whether the action is relevant.* Emotions can secondly, however, create a disruption that is necessary for sensemaking to take place. Some participants therefore point to the importance of using emotions positively to manage unforeseen events and to create new knowledge structures.

In sum, the findings of research question two show that both positive and negative emotions can play a central role in the ability to adapt and respond to unforeseen events. The participants consider emotions to directly or indirectly influence the other main themes. The findings finally indicate that it is important to analyze emotions at all levels, as they can have a significant effect on sensemaking and performance.

## Discussion

The purpose of this study is to facilitate the better targeting, selection and development of competence at the individual, social and organizational level to enhance preparedness to face the unforeseen. The findings are based on the responses of individuals who have personal, extensive and unique professional experience of unforeseen events in a national and international context. Theory and empirical studies indicate that it is possible to prepare for unforeseen events. This study is, however, the first to publish in-depth information provided by recognized experts from different sectors and organizational levels, on the particular competence required to comprehend and manage these events. Our main finding was that organizations should build relational types of competence that are based on an adaptive strategy and the concept of resilience.

This study had two aims. One was to explore the experience of highly knowledgeable professionals of unforeseen events at the operative, operational and strategic level, to promote understanding. The other was to explore what resilient competencies can be beneficially applied in organizations to enhance performance, irrespective of the scenario or event that occurs. This study contributes to prior knowledge in three ways.

First, the findings, through revealing insights into rich practical experiences, extend current theoretical research on the unforeseen. These insights bring substantial nuances and increased understanding to the phenomenon. Second, the findings also mirror the findings of previous research into factors that underpin competence for the unforeseen. Participants experienced interaction, social support, general self-efficacy, concurrent learning and improvisation as being essential core competencies. This study, however, clarifies, adds nuance to and provides novel information to the understanding of these factors and relationships, so allowing previously identified competencies to be better targeted for educational purposes. Third, new competencies were presented. The findings therefore suggest that the notion of traditional rational and cognitive qualities should be expanded to include the role emotions play in organizational life.

This study presents main types, units and elements of competence that may help in preparing individuals, groups and organizations for unforeseen events, and therefore clarifies, supports and extends existing research.

### Main Findings and Implications

The findings of this study lead to the proposal that organizations should build wide-ranging relational and resilient competencies to comprehend and manage the increasingly complex nature of demanding events. This study, based on the two research questions, suggests three main areas of implications.

#### The Complex Unforeseen Phenomenon

The definition and recognition of the unforeseen seems to be paramount to an organization’s understanding of preparedness and readiness – from the strategic to the operative level. This has both practical and theoretical implications. The term relative is used in the definition of the unforeseen, because there are different degrees of the unforeseen. For example, the known versus unknown, unforeseen phenomenon occurring suddenly and surprisingly when unknown. Whether risks are then identified, and if identified are acted upon or ignored, determines the degree to which an event is cogitated as unknown.

Experience is also a central concept of the definition, which implies that an unforeseen event has an objective and a subjective dimension. An event can therefore be experienced as having different degrees of unforeseenness, the degree depending on factors such as the person, the experience, training, location, time, extent of identified warning signals, and type of event and the degree of known versus unknown for the person (Torgersen, 2018b).

The findings of this study furthermore point to weaknesses in established linear planning models for learning and training for unforeseen events. Conditions are not the same, one does not know what one is facing, or the specific competencies the event requires. This is a fundamental and significant problem for many organizations. An important element of preparedness for the unforeseen is therefore determining the basic and resilient competence required under such conditions. This will allow traditional learning and competence goals to be complemented with new and more adaptive plans and dynamic models for learning and training, e.g., SECI-model (Nonaka et al., 2000).

The ability to move beyond the somewhat overwhelming experience of temporary shortcomings may have a fundamental impact on the outcome of an event. The findings show that the experience of the unforeseen as a phenomenon depends on context, time and perspective. Phenomena are dynamic and in constant motion, and most participants therefore find it difficult to define an event as completely unforeseen. It might theoretically and objectively be possible to eliminate the unforeseen as a phenomenon, especially when reviewing an event in hindsight. Whether or not an event is defined as unforeseen is, however, not of relevance to those who have to deal with it. This study shows that it is the subjective experience that has real practical significance. Recognizing the unforeseen as a phenomenon is therefore pivotal. For example, holistic and strategic forecasting analysis could contribute to understanding trends and exploring the implications of change (Kuosa, 2016). This might enhance adaptive strategic planning and help avoid nearsightedness and cognitive closure around important developments in our environment. Having the ability to think “outside the box” when considering less likely and tenable but potentially decisive ruptures or events, is therefore important. It is impossible to predict exactly what will happen in the future. A range of possible developmental paths can, however, be identified. This study therefore suggests that organizations should adopt an interdisciplinary approach, which balances traditional emergency preparedness and risk analysis with measures from crisis management and the concept of resilience.

#### Social and Relational Types of Competence

The participants considered social and relational factors such as interaction, leadership and social support to be essential competencies for the unforeseen. The findings also show that there is a conceptual link between these units of competence and similar relational processes such as trust, communication, psychological safety, intention and value-based leadership. This is in line with current literature, which emphasizes that organizations are open and complex social systems in which people affect each other and interact with the environment (Jacobsen and Thorsvik, 2019; Edmondson and Lei, 2014; Daft, 2015). It seems that these relational processes represent a way of coping with and balancing the uncertainties that unforeseen events involve, through reducing the need for control and by encouraging decision-making and empowerment. Further research is, however, needed to more thoroughly investigate these conceptual relationships and their significance.

This study at the same time shows that individual-level differences in characteristics and qualities can be beneficial, and therefore desirable, as they provide competence diversity and flexibility in complex task solving. The analysis, even so, showed that those who seem to deal with unforeseen events better, share commonalities of individual-level characteristics. The findings therefore show that individual differences are multi-faceted. This study proposes that these differences should be viewed in a relational, situational and contextual perspective, within the framework of an organization and a culture. Future research may, however, benefit from including more individual-level factors in the study of competence for the unforeseen, as this can lead to improvements in recruitment, selection procedures in leadership and team-development.

The findings show the ability to reflect and learn to be key elements in the creation of new knowledge structures and new ways of acting. The analysis, however, raises the question of the prerequisites for this, in particular how two factors such as the time perspective and proximity to the situation can affect the outcome. Concurrent learning was a new concept to many of the participants. They were more accustomed to the traditional approach of experiential learning (Kolb, 1984). All participants, even so, assessed this type of situated learning (Lave and Wenger, 1991) and reflection-in-action (Schön, 1992; Yanow and Tsoukas, 2009) within a community of practice (Wenger et al., 2002) to be important and sometimes decisive to the outcome. The experts stated that it is particularly important that leaders actively create space for reflection, sharing and learning. This in practice involves the application of the emotional and mental capacity to stop, to capture relevant information and details, to communicate and ask critical questions about the situational understanding and effectiveness of measures. It is important to stay attuned to the situation and be open both to surprise and the possibility of publicly “not knowing.” This will ensure that one always strives to be in a process of learning (Yanow and Tsoukas, 2009) and exploration (March, 1991), individually, socially and culturally (Antonacopoulou and Chiva, 2007).

There seems to be a close link between the ability to learn along the way and creative processes and behavior. The participants highlighted that the ability to improvise at all levels can create new and other ways of solving challenges. The findings of this study are therefore consistent with the argument that improvisation can be as important at an organizational level as at an individual level (Hadida et al., 2015). Some participants, however, pointed out that the ability to improvise can be more directly expressed at an operative and practical level. The findings also support the view that improvisation is a mechanism that operates between flexibility and structure and exploration and exploitation (Vera and Crossan, 2005). The ability to improvise can, however, be tacit. It is often distracted by structure, planning, standard operating procedures (Weick, 1998) or the fear of making mistakes.

All the participants in this study furthermore believe an important precondition for the aforementioned competencies is that an organization has a minimum of general preparedness for the most likely scenarios. This includes a mental readiness, necessary equipment, and applicable planning processes and emergency plans. Varied practice and training with relevant actors are also important. This study proposes that this becomes even more important in a complex operational environment which is more and more transboundary in nature. A more adaptive approach to emergency preparedness and contingency plans is therefore needed.

#### Emotion Efficacy

The findings show how affective phenomena can influence perception, cognition and behavior when experiencing an unforeseen event. Such events impose abrupt and often unexpected change upon people and organizations. The affective state therefore orients them to respond. This study clearly shows that it is the capabilities and competences of the employees of the entire organization, including all their cumulative senses, physical reactions, emotions, and cognition, that encounter such an ongoing event. Researchers often base their definition of emotions on one or two of these components (Vikan, 2014). What is meant by emotion, affect and by related terms such as emotional traits, sensory experience and mood, however, remains a challenge. A consensus is developing that provides some clarification of the relationships between them (Keltner and Lerner, 2010; Forgas, 2014). Emotions are assumed to be briefer, more intense, and to usually have a cognitive content and informational value. This study shows that emotions are also presumably more context specific and are more focused on a particular cause, intentional object or environmental occurrence (Frijda, 1986, 2007; Keltner and Lerner, 2010). Emotions are also viewed as being a complex, relational and multidimensional phenomenon. Professional experts, when telling about unforeseen events or situations, described bodily changes, something they perceived or thought of, and something they did or thought to do. This shows that emotions are made up of different components. This study therefore regards emotions to be; *a coordination of experience, bodily reactions and behavior. Emotions involve work (energy consumption) in the form of bodily (physiological) revival, leading to selective drive or selective motivation, with specific experiences and forms of behavior* (Vikan, 2014, p. 17).

Unforeseen events create powerful emotional experiences that demand individual, social and situational adaption and adjustment. The likelihood of inconsistencies and incongruence between goals, objective and rational reality, and the understanding that is formed in the consciousness of employees therefore increases. This sheds light onto the importance of the subjective experience and the perspective of those who must manage an unforeseen event, when considering a casual attribution for the event, potential responses, and future consequences of different actions (Folkman and Lazarus, 1989). A condition that is perceived *in situ* to be chaotic and complex, normally raises the intensity of emotions and profoundly affects tolerance of negative emotions, level of stress, and cognitive processes (Forgas, 1995, 2001; Weick et al., 2005; Maitlis and Sonenshein, 2010; Maitlis et al., 2013; Hannah and Parry, 2014).

The findings furthermore show the potential of stimulating and exploiting positive emotions in dealing with an event, and so also the motivation of associative, creative and broadening patterns of attention and orientation (Isen, 1987; Fredrickson, 1998, 2001; Clore and Robinson, 2012). The importance of emotions in activating positive memories, cognitive resources, and encouraging relational processing makes them particularly important (Clore and Robinson, 2012, p. 327). Emotions can, in turn, motivate adaptive individual and collective actions that facilitate adjustment and change, and promote resilience to the potential stressors and trauma that unforeseen events trigger (Fredrickson et al., 2003). For example, the emotion knowledge and the rich metaphorical expressions (Kövecses, 2003; Niedenthal, 2008) of the professional experts in this study imply that emotions can play a central role in the ability to tolerate, adapt and respond appropriately to unforeseen events.

Emotions therefore have the potential to generate negative consequences and positive opportunities, depending on the valence of their experience (Russell, 2003). Emotions can, on the one hand, be a source of fear, anxiety, dread, worry and insecurity, which may reduce individuals’ and groups’ cognitive capacity and sensemaking (Mineka and Sutton, 1992; Mathews and MacLeod, 1994; Maitlis and Sonenshein, 2010; Hannah and Parry, 2014). The findings similarly show that negative emotions can have an inhibitory and negative effect on social interaction within and across organizations and sectors in the face of transboundary unforeseen events. If the emotional state is negative, then it is more likely that people and organizations will categorize other stimuli in congruence with the current emotional state (Niedenthal and Setterlund, 1994; Niedenthal, 2008). Positive emotions such as surprise, excitement, amazement, astonishment, interest and optimism can, on the other hand, release positive energy and mobilize resources for insightful patterns of thought, problem solving, judgment and decision-making (Fredrickson, 2001; Sandberg and Tsoukas, 2015; Shin, 2015).

The professional experts in this study have, in total, experienced how emotions are influenced by relational processes, the ability to notice and to extract important cues and learning processing. This comprises the need for psychological safety, critical thinking and innovative behavior (Edmondson, 1999). The reflections of the majority of the participants latently showed, however, a somewhat overstated focus on the rational and the cognitive. They all manifest or latently expressed the opinion that moving quickly into the cognitive domain is important to functioning well. Emotions can therefore assume the role of being something that is separate and disruptive, dysfunctional and negative in work in which behavior is viewed as being distinctly rational. This study, however, contributes to the breaking down of traditional boundaries between cognition and emotion (Keltner and Lerner, 2010), and is in line with the increasingly acknowledged findings that emotions and cognitive capacity are inextricably linked. The findings propose that emotions influence many cognitive processes such as attention, risk perception, situational awareness, sensemaking, judgment, problem-solving, creative behavior, learning, decision making and human interaction (Maitlis and Sonenshein, 2010; Breakwell, 2014; Sandberg and Tsoukas, 2015; Shin, 2015).

Emotions can therefore generate and motivate positive adaptive actions, which can facilitate change when dealing with unforeseen events. McKay and West (2016) concept of emotion efficacy provides one suitable approach to the articulation of competence implications. Emotion efficacy is defined as; *how effectively a person, group or organization can experience, exploit and respond to a full range of emotions in a contextually adaptive, favorable and value-based manner* (based on McKay and West, 2016). Research on emotion efficacy, emotional competence, emotional regulation, emotional intelligence and similar concepts is, however, inconsistent on the effect these have on work-related outcomes (Salovey and Mayer, 1990; Mayer et al., 2008; O’Connor et al., 2019). More research is needed. This study is, however, in line with findings that indicate that competence on emotional qualities can give a substantial benefit in many situations (Keltner and Lerner, 2010; Hannah and Parry, 2014; Shin, 2015). This may, for example, be particularly true in emotionally laborious and demanding work such as the fire service, health care, the police and the military (O’Boyle et al., 2011). These organizations should therefore seek to develop more knowledge and competence on the role and influence of emotions at the individual, dyad, group and organizational or cultural level (Fischer and Manstead, 2008; Ashkanasy and Humphrey, 2011). This study, therefore, in line with the growing volume of research on emotions, contributes to the broader implication of emotions and intra and interpersonal processes in institutional processes (Lok et al., 2017; Goodrick et al., 2019). The findings show that it is important to analyze emotions at all levels, as they clearly have a significant effect on human performance, particularly when surprise and uncertainty are involved and there is a need for social interaction, learning and creative behavior. *Emotion Efficacy* is therefore considered to be one of the main types of competence for the unforeseen. This finding points to a gap in the current literature, which has primarily focused on rational and cognitive aspects, and on individuals extracted from the social context.

### The Resilience Competence Face Framework of the Unforeseen

**FIGURE 3:**
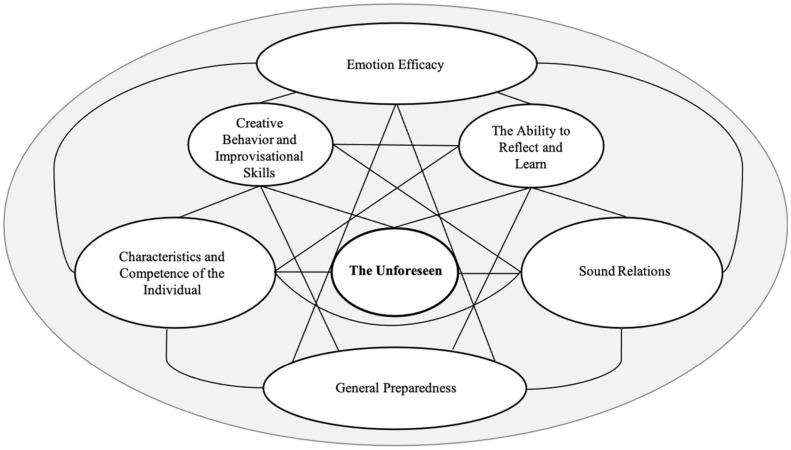
The Resilience Competence Face Framework of the Unforeseen© showing the six main types of competence.

An interplay between the types of competence, and the dynamics of competencies at the individual, group and organizational level emerged across the data. Figure 3 integrates the main findings of this cross-disciplinary and multilevel study and presents a relational framework of resilient competence for the unforeseen at an elevated and generic level.

The framework shows the particulate competency that interact from the individual level to the social and organizational level. The six main types of competence are presented as parts of a whole that respond to the unforeseen phenomenon in the center. No attempt has, however, been made to separate cause from effect, the factors having a linear inter-relational association, a change in one factor affecting the other factors. The context of the unforeseen is a complex, unknown and unpredictable environment, and it is therefore difficult to predict the relationships and their strength. The framework, however, inspires further research and the adoption of a variety of levels of analysis or explanations and introduces a way to organize complex data.

### Strengths and Limitations

The generic qualitative inquiry of this study allowed subjective reflections and opinions to be directly gained from the perspective of 13 highly experienced practitioners on real-world events (Caelli et al., 2003; Kahlke, 2014; Percy et al., 2015). Adopting this research approach and using the framework of thematic analysis also provided an iterative and systematic closeness to the rich dataset during the coding process (Ritchie and Lewis, 2003; Percy et al., 2015). Coherent themes and sub-themes emerged, the participants’ experiences being accurately described as meaningful (Braun and Clarke, 2006). Competencies for the unforeseen were therefore revealed and recognized, better insight into the complex relationships between different types of competence and the underlying elements were obtained, and a deeper understanding of the complex unforeseen phenomenon was gained (Morse and Richards, 2002).

The limitations of this study should, however, be acknowledged. Firstly, a single data source does have its limitations. Even so, data from in-depth qualitative interviews of professionals were considered beneficial when participants could not be directly observed in a natural field setting. The highly informed participants were also able to provide substantial and rich historical information. Initial quantitative data results from previous studies could be further explained with qualitative interview data (Creswell and Creswell, 2018). In addition, there were sufficient participants to achieve saturation. Despite this, reliance on self-reports is retrospective, and the data collected can be subject to social desirability bias and memory concerns, including overconfidence, memory construction and forgetting (Myers and DeWall, 2018). Building trust with the participants, based on an unassuming understanding of the context and the topic of this study, were emphasized. A structured data analysis strategy of repeated leaps into the data, co-analysis, and comparisons led to a deeper understanding of the complexity of the phenomenon and all that the body of data was compromised of. Potentially disconfirming data was simultaneously searched for. This was a particularly important aspect in letting themes emerge directly from the data, instead of from the previous research and theory that guided parts of the interviews. The interviews were also conducted sequentially to the point of redundancy, where no new concepts, themes or insights emerged (Lincoln and Guba, 1985; Trotter, 2012).

Secondly, the single interviewer and the researcher’s horizon of understanding (Rennie, 1994) and subjectivity could interfere with a just collection and interpretation of the data, and therefore introduce the possibility of researcher bias. The first author’s background is from various positions in the defense sector. Therefore, this study was approached with previous knowledge and involvement in the topic and associated presuppositions. This could also affect interaction with the study participants (Morrow, 2005). Important precautions such as consulting and discussion with other researchers, colleagues or practitioners, perspective from different participants and previous data, external auditor of transcripts, participatory consciousness, keeping an audit trail, memo-writing and reflexivity were taken to manage subjectivity and to enhance validity (Creswell and Creswell, 2018). Transparency and documentation throughout the data collection and analysis of data were emphasized. The effectiveness of these measures cannot be clearly assessed (Morrow, 2005). The first author’s background has, however, been a source of advantageous pre-knowledge when asking for clarification or delving more deeply into the participants‘ experiences and for staying close to the data. The pre-understanding of the single interviewer therefore made the in-depth interviews a consistent, mutual and trustful conversation between equals (Brinkmann and Kvale, 2015). The participants were also involved throughout the interview-process and were asked, for example, to pick a time and location for the interview themselves, so that they could feel free to talk openly (Sandelowski, 2002).

Thirdly, the experts in this study were from organizations that are primarily hierarchical and are dominated by men. This study does not, therefore, take into consideration how unforeseen events would be impeded in less hierarchically enforced structures or cultures of more feminine relationships. Our sample mainly consisted of people with extensive leadership experience. The views of the professional experts could therefore differ from those of younger and more inexperienced persons, in other functions and roles, and persons from other cultures. The transferability of this study’s findings to other cultures and organizations is therefore questionable (Morrow, 2005). This study has, however, obtained a broad cross-sectorial representation of relevant organizations and eminent participants. All were selected based on specific criteria (e.g., professional experience and socio-demographics) to provide a range of views, information-rich data from unforeseen events, and the situational diversity necessary for reaching data saturation and identifying thematic patterns (Hennink et al., 2011). Different positions and roles at a range of organizational levels were also represented to achieve maximum variety. The participants’ unique expertise and extensive experience of unforeseen events are valued to be genuinely acknowledged. The findings presented in this study are therefore considered to be parallel to those of other people and organizations that experience unforeseen events.

Future studies may address the limitations of this study. Researchers could, for example, consider using quantitative approaches and techniques to capture the bodily-emotional-cognitive-behavioral linkage. They may also collect information from multiple sources and subjects holding different levels of expertise, from different organizations and from different cultures. Future studies may use the findings of this study to further develop measurement tools for evaluating performance for the unforeseen and the particular types of resilience competence. The effectiveness of selection and training interventions that are based on the findings of this study may furthermore be tested and implemented. A promising way forward could be emotion efficacy, creative behaviors such as improvisational skills, concurrent learning abilities, and the relational qualities in effective leadership and social interaction.

## Conclusion

This study proposes that it is possible to prepare and adapt to unforeseen events. It has explicitly, using in-depth interviews with professional experts and thematic analyses based on a generic qualitative approach, shown that competence in the emotional, relational and interpersonal makes individuals and organizations more resilient.

The study has important practical implications. These include implications for the ability to recognize, regulate and use emotions constructively, but also for general self-efficacy and the ability to socially interact and make decisions in dilemma situations based on concurrent learning and improvisation. A social environment built on trust, psychological safety, emotional and moral support is also of great importance, in which an adaptive and beneficial learning culture where an acceptance of failure and a desire to learn can be created. This study shows that these competencies are the most important that leaders and organizations can develop to manage unforeseen events. Preparedness, relevant contingency plans and planning-processes, emergency exercises, equipment, and effective routines and processes are also important and fundamental. Our findings, however, show that relational and social factors seem to be even more important when unforeseen events actually occur.

This study implies that future events will come more quickly, more discontinuously, give more complex operational environments and side-effects, and be less predictable than in the past. As a result, the pace at which individuals and organizations must adapt to changing circumstances and unforeseen events has increased.

This study therefore suggests that the *Resilience Competence Face Framework of the Unforeseen* (see Figure 3) could be applicable across different organizations and contexts. The framework is potentially suitable for guiding the targeting and development of resilient competencies of support persons, groups and organizations that face unforeseen events. Further research, however, is needed to explore the framework across different organizations, cultures and contexts. Finally, the relations between the main type of competence, units of competence and elements of competence need to be further examined.

## Data Availability Statement

The dataset for this manuscript is not publicly available because of the sensitive information it contains regarding participants’ anonymity. Requests about the dataset should be directed to MH, marius_herberg@hotmail.com.

## Author Contributions

Both the authors designed and adopted the study. MH conducted the data collection. MH and G-ET decided upon the research questions and conducted the analysis. Both the authors revised the manuscript. MH wrote the manuscript. Both the authors discussed and commented on the final version of the manuscript. Both the authors agreed to be accountable for all aspects of the work and have read and approved the final version for publication.

## Conflict of Interest

The authors declare that the research was conducted in the absence of any commercial or financial relationships that could be construed as a potential conflict of interest.
